# The Desire of Medical Students to Integrate Artificial Intelligence Into Medical Education: An Opinion Article

**DOI:** 10.3389/fdgth.2022.831123

**Published:** 2022-05-13

**Authors:** Timothy C. Frommeyer, Reid M. Fursmidt, Michael M. Gilbert, Ean S. Bett

**Affiliations:** ^1^Boonshoft School of Medicine at Wright State University, Dayton, OH, United States; ^2^Ohio University College of Osteopathic Medicine, Columbus, OH, United States

**Keywords:** artificial intelligence, machine learning, medical school, precision medicine, drug discovery, diagnostics, healthcare administration, curriculum

## Introduction

Medicine is at the precipice of change. The advancement of artificial intelligence (AI) and machine learning (ML) algorithms are reshaping the way physicians and healthcare providers approach the practice of medicine. In recent years, AI has rapidly evolved into applicable medical technology geared for clinical practice. These systems are now processing increasing amounts of complex data, improving the viability of wearable biometric devices, optimizing the use of diagnostic algorithms, and utilizing pattern recognition within large datasets such as electronic health records (EHR) (1–4). The sheer speed and efficiency of these systems has the potential to outperform physicians in specified tasks, which could allow more time for physicians to do other important work, such as engaging with patients in deliberate counseling and education, as well as addressing health inequities domestically and abroad.

As with many innovations, there has been resistance from physicians and healthcare workers with the expansion of AI technologies. The lack of comprehension, the potential administrative load, the lack of a legal framework, and the fear of job security have all contributed to this opposition (5–8). Regardless of the current divide on the perception and utility of AI, we believe its adoption in clinical practice is inevitable due to the incentives of the healthcare business sector and the improvements it will provide in patient care. Technology giants including Google and IBM are investing in AI technology for mining medical records (9). Additionally, start-ups such as Enlitic are using deep-learning (DL) algorithms to interpret medical images significantly faster than the average radiologist, providing radiologists with the ability to accomplish other tasks and evolve their roles to enhance patient care (9). AI's integration within medicine is unavoidable given that big-business and start-ups alike are developing technologies enabling more effective and efficient medical care. Therefore, it is imperative for the medical community to become leaders by guiding the integration of AI, ensuring these technologies enhance health outcomes and provide a more equitable distribution of patient care. As outlined in this manuscript, we believe the best place for this change to begin is within medical schools. Members of our team have prior experience in precision medicine, drug discovery, diagnostics, and hospital administration, which we use to provide a unique perspective on how AI will be integrated into the medical profession. As future physicians, our team calls for the integration of AI curriculum within medical education.

## The Current Landscape

### Precision Medicine

Precision medicine offers future clinicians an opportunity to implement new and innovative strategies to deliver healthcare. One member of our team worked with AI systems that created a therapeutic and personalized regimen for cancer patients. In this setting, computers were trained how to analyze pathology reports, progress notes, and relevant clinical data to develop a unique patient profile. The trained AI algorithm would then utilize this profile, which includes the patient's type of cancer as well as where they are in treatment to match the individual to an optimized clinical trial. Moreover, the system allowed doctors to identify patients with similar profiles to the patient being treated, allowing oncologists to have a better understanding of what options were available in different healthcare networks. These technologies also empower patients by affording them more control over their own treatments and providing more personalized care. Ultimately, the challenge of this approach and data aggregation at this scale is mainly limited by the vast amount of processing power required for its execution. Nevertheless, these procedures, as well as the ecosystems built around AI and precision medicine, are beginning to have real world clinical outcomes (10–12). Thus, it is important for medical students to become more familiar with AI systems because of its expanding foundation in the implementation of precision medicine.

### Drug Discovery

Precision medicine utilizing AI may offer a solution to another growing challenge facing clinicians: therapy-resistant patients. One novel way clinicians can combat these resistant patient populations is through the development of in-house drug screening programs for patient-specific therapeutic discovery. High-content screening has been a staple in the pharmaceutical industry, but its real-time implementation for drug discovery is relatively new due to faster computational processing and AI technologies. High-content screening that is high-throughput allows researchers to investigate phenotypic patterns at a cellular level with increasingly large feature sets that capture hundreds to thousands of cellular characteristics across, potentially, millions of cells (13). This necessitates the use of AI algorithms to allow future clinicians to screen therapy-resistant patients and compare them to FDA approved drugs as well as orphaned agents. Recently, a team from the University of Michigan was able to screen COVID-19 infected cells against every FDA approved drug and natural product libraries to uncover existing therapeutics that could be utilized in clinics to treat COVID infected patients awaiting vaccines (14). While this strategy may not be a panacea for every patient's disease, it certainly offers a chance to enrich the lives and outcome for patients. Consequently, as AI continues to enhance high-content screenings, physicians will need a better understanding of these technologies in order to make better clinical recommendations and improve their decision-making.

### Diagnostics

AI technologies offer enhanced capabilities of disease detection and prognosis, particularly in medical fields like radiology and pathology. Medical school offers fundamental insight into the underpinnings of histology and radiology, but the current curriculum fails to showcase how AI technologies will enhance these fields and alter the current practice. In light of this changing landscape, medical students should be offered insight so that they are better equipped within these evolving practices. For example, histological and radiological imaging can be condensed into data sets that may be exploited by AI (15). These systems are able to develop new ways to identify patterns indicative of disease, such as computational pathology (CPATH). Unlike a pathologist, CPATH goes beyond human perception and is better equipped to utilize the spatial recognition of histological slides (16). Moreover, CPATH research has unveiled new morphological characteristics that offer more specific grading for Ductal Carcinoma *in situ* (DCIS) and have found novel stromal features that yield independent prognostic information about breast cancer progression. There are similar developments in the field of radiology, which identify pathologies such as intracranial hemorrhages and strokes as well as workflow optimization for radiologists (16–18). Similar to precision medicine, the advent of new diagnostic AI technology enhances physician capabilities and paves the way for faster disease recognition and improved patient outcomes.

### Healthcare Administration

The AI-based advances in precision medicine, drug discovery, and diagnostics represent only a portion of the potential benefits of AI's integration within medicine. Another significant advantage is the impact it will have in healthcare administration. The current practice of medicine is riddled with unnecessary complexity, leading to increased costs and driving clinician burnout (19). As a result of the significant administrative burden, physicians are spending less time with patients and facing increasing workloads (20). Patients may receive substandard care because every hour a physician spends with a patient yields an additional two hours of work in EHRs, which may result in diminished personalized and deliberate interactions (20). In 2020, Khairat et al. found excess use of electronic health records was directly associated with increased physician burnout and higher rate of medical errors (21). The current physician-patient interaction is characterized by data entry, not data analysis, resulting in inefficient medical care, diminished patient outcomes, and increased healthcare cost (13, 22–24). Although still in their early stages, AI systems show promise in enhancing clinical diagnosis and decision-making, as well as allaying the significant documentation burden contributing to physician burnout (25–27). In order to optimize quality and patient safety, healthcare organizations must advance a shift toward AI-enhanced practice. This transition could be marked by improved physician workloads, automation of data entry, augmented decision making, streamlined workflows, and more time spent speaking directly to patients. The end result would be an upgraded healthcare system characterized by clinicians collaborating with AI-enabled modalities, resulting in more accurate and patient-centric care. The outcome would also enable the next generation of physicians to allay the burden of medical burnout, leaving the door open for innovation and optimized patient care.

## Call to Action

Medical schools provide the optimal environment for the modification of physician attitudes and beliefs. As presented by the American Medical Association, the current state of AI curriculum is lacking, with only select medical schools reporting AI initiatives (28, 29). Given the lack of education in digital medicine, medical schools need to start incorporating education about AI and its utility within healthcare into an upgraded curriculum. We suggest guided seminars and courses on biostatistics, digital health literacy, and engineering technologies. Given the time constraints of medical school, these courses may benefit from being offered initially as elective and supplementary to the existing foundational curriculum. By enacting a bottom-up approach, the acceptance of AI would gradually encompass the totality of healthcare, allowing physicians to guide the use of augmented medicine. We believe brevity is key to this transition due to technology's rapid and exponential evolution. Medical professionals must embrace the promise and challenges of AI-powered technology and augmented medicine, which could result in optimal outcomes for both the physician and patient alike. If we act now, a more educated and prepared coalition of students and physicians will be better equipped for the fast-changing medical landscape, leading to future physicians who will be more competent, inventive, and compassionate in the medicine of tomorrow.

## Author Contributions

TF, RF, and MG contributed equally to this work and wrote the first draft. EB helped conceptualize the work and edited and provided further input. All authors contributed to the article and approved the submitted version.

## Conflict of Interest

The authors declare that the research was conducted in the absence of any commercial or financial relationships that could be construed as a potential conflict of interest.

## Publisher's Note

All claims expressed in this article are solely those of the authors and do not necessarily represent those of their affiliated organizations, or those of the publisher, the editors and the reviewers. Any product that may be evaluated in this article, or claim that may be made by its manufacturer, is not guaranteed or endorsed by the publisher.
